# Hardware/Software Co-Design of Fractal Features Based Fall Detection System

**DOI:** 10.3390/s20082322

**Published:** 2020-04-18

**Authors:** Ahsen Tahir, Gordon Morison, Dawn A. Skelton, Ryan M. Gibson

**Affiliations:** 1 School of Computing, Engineering and Built Environment, Glasgow Caledonian University, Glasgow G4 0BA, UK; Gordon.Morison@gcu.ac.uk (G.M.); Ryan.Gibson@gcu.ac.uk (R.M.G.); 2 Department of Electrical Engineering, University of Engineering and Technology, Lahore, Punjab 54890, Pakistan; 3 School of Health and Life Sciences, Glasgow Caledonian University, Glasgow G4 0BA, UK; Dawn.Skelton@gcu.ac.uk

**Keywords:** fall detection, wearable sensors, classification, machine learning, fractal features, hardware software co-design, FPGA, reconfigurable design, embedded system on chip

## Abstract

Falls are a leading cause of death in older adults and result in high levels of mortality, morbidity and immobility. Fall Detection Systems (FDS) are imperative for timely medical aid and have been known to reduce death rate by 80%. We propose a novel wearable sensor FDS which exploits fractal dynamics of fall accelerometer signals. Fractal dynamics can be used as an irregularity measure of signals and our work shows that it is a key discriminant for classification of falls from other activities of life. We design, implement and evaluate a hardware feature accelerator for computation of fractal features through multi-level wavelet transform on a reconfigurable embedded System on Chip, Zynq device for evaluating wearable accelerometer sensors. The proposed FDS utilises a hardware/software co-design approach with hardware accelerator for fractal features and software implementation of Linear Discriminant Analysis on an embedded ARM core for high accuracy and energy efficiency. The proposed system achieves 99.38% fall detection accuracy, 7.3× speed-up and 6.53× improvements in power consumption, compared to the software only execution with an overall performance per Watt advantage of 47.6×, while consuming low reconfigurable resources at 28.67%.

## 1. Introduction

Falls are the highest cause of death from injury in individuals over the age of 65, resulting in high mortality, morbidity and immobility [1]. Falls produce a high cost for the National Health Service (NHS), with associated costs over £2 billion and 4 million bed days per year [1]. Fall Detection Systems (FDS) provide aid and support for individuals who live alone and may not be able to call for prompt medical aid due to injury or unconsciousness. Additionally, fall detection systems have been reported to obtain 26% and 80% improvements for hospitalization and death rates by providing immediate medical aid on fall event detection [2]. FDS detect and classify falls from other movements of a human body caused by Activities of Daily Life (ADL).

The movements of a human body are a result of complex non-linear interactions between feed-forward spinal circuitry and feed-back mechanisms from muscles, skin and various senses [3]. It is a non-linear dynamic system, which can be modelled as a chaotic system and analysed with chaos theory and fractal dynamics. While, the present work in non-linear dynamic systems understands the human body as a chaotic system and is limited to walking activity and gait analyses. There is a significant gap in understanding of the non-linear dynamics of falls through fractal analysis of accelerometer signals for falls and ADL.

Fractals are self-similar structures where the whole is similar to its parts. Self-similarity in structures or patterns can be approximate and limited to one or more parts. Self-similarity can also be statistical in nature. Many real world objects show statistical self-similarity where statistical properties are similar at different scales. Statistical self-similarity may manifest in time varying signals. However, sensor signals do not need to be truly fractal in nature. The fractal dimension has a positive correlation with irregularity of a signal, according to Mandelbrot [4] and can be used as a measure of signal irregularity. Our work first determines if such an irregularity measure of the signal is a discriminant feature for classification of falls from ADL. While many fractal analysis methods have been proposed in literature, our work utilises Autoregressive Fractionally Integrated Moving Average (ARFIMA) for fractal analysis, since the technique has the advantage of solving the problem of biased overestimation and higher errors of fractal parameters for complex processes [5,6], such as human movements and activities. ARFIMA works with stationary signals and non-stationary signals can be analysed with ARFIMA by first conversion to stationary signals [7]. Once the signal is converted to a stationary signal, ARFIMA is applied and fractal parameters are calculated. The fractal parameters are then determined for the original non-stationary signal through conversion from the stationary signal parameters [7]. Therefore, stationarity testing is imperative for such an approach. For stationarity testing fall and ADL signals are analysed with Augmented Dickey-Fuller (ADF) and Kwiatkowski-Phillips-Schmidt-Shin (KPSS) tests in Section 4.2 for a rigorous treatment to understand the stationarity characteristics and provide empirical evidence of non-stationarity. For non-stationary signals it is important to find the order of difference for conversion to a stationary signal. ARFIMA modelling is performed in Section 4.3.1 using a public fall dataset by Kwolek et al. [8] discussed in Section 3. ARFIMA modelling in Section 4.3.1 shows that fractal parameters are useful discriminant features to classify falls, to the best of our knowledge, fractal parameters have not been used to classify falls from ADL. While, ARFIMA provides robust analysis, it requires development of ARFIMA models and careful inspection of goodness of fit functions to determine the fractal dynamics, which is not appropriate for real-time classification events. The relationship of Discrete Wavelet Transform (DWT) with fractal computations [9,10] is leveraged for real-time implementation on an embedded wearable device for fall detection.

Hardware reconfigurable design of a fall detection system can satisfy the design constraints of high performance per Watt to enable real-time implementation with computationally intensive algorithms for higher accuracy requirements. Embedded multicore FPGAs are a suitable platform for hardware implementations and acceleration of intelligent systems/machine learning models on embedded devices and provide good performance to power ratio [11,12]. A real-time sustainable operation requires low power consumption and high throughput with high performance per Watt for a multi-level wavelet transform and fractal features extraction process for FDS. The high throughput and low latency achieved with reconfigurable design can provide system scalability for multiple multichannel sensors at a low power budget. One of the major contributions of our work is a reconfigurable accelerator design which leverages multi-level DWT for real-time fractal computations performed directly on non-stationary signals, since ARFIMA method is not feasible for an embedded low latency implementation. We utilise a hardware/software co-design of a fall detection system to satisfy the design constraints of high performance per Watt to enable real-time implementation of computationally intensive parts of the algorithm. The hardware accelerator for multi-level DWT and fractal features leverages various design innovations and optimizations. Pipelined arithmetic trees are utilised for convolution operations and variance computations. Memory system optimizations are performed to provide required throughput to the arithmetic trees through cyclic two-port memory blocks. DWT convolution and subsequent downsampling operations are optimised into a single operation by skipping alternative computations and storing filter coefficients in flipped form. The number of clever design optimizations and their results are discussed in Section 7.1 and Section 8, respectively. The fractal dimensions along with the DWT low pass coefficients obtained as a byproduct of fractal computations from the hardware accelerator are passed on as features to the embedded ARM core for machine learning classification. A Linear Discriminant Analysis (LDA) classifier is then applied to the feature set for classification of falls on an embedded ARM core in the SoC device. We show that with fractal features and DWT coefficients, classification of falls from ADL provide high accuracy of 99.38%, a high throughput, low power consumption and high performance per Watt for a hardware/software co-designed system. The main contributions of our work are as follows:

- Fractal analyses undertaken to explore the irregularity of accelerometers for stationary and non-stationary fall/ADL signals.
- Fractal features utilised as irregularity metric of the signal for fall detection and classification for the first time, to the best of our knowledge.
- Energy efficient hardware accelerator for high throughput and maximal re-use of computation blocks for the feature extraction process.
- Multi-level DWT and fractal features clever design innovations and optimizations, including pipelined arithmetic trees for fractal computations, cyclic two-port memory optimizations, convolution and subsequent downsampling optimization through a single operation etc.
- Hardware/software co-design of a portable FDS system for sustainable operation and real-time classification, evaluating wearable accelerometer sensor.
- Higher performance and accuracy of 99.38% than existing hybrid vision and accelerometer fall detection systems.
- Low power and latency optimised design with high performance per Watt of 46.7×.

## 2. Related Work

Current fall detection systems have a significant focus on using machine learning algorithms and can be classified into sensor-based [13,14,15,16,17] and vision-based [18,19,20,21,22,23] systems. The data obtained from sensors or camera is processed to extract features for classification of human movements as falls. Most sensor-based FDS have employed acceleration data [8,16,24,25]. Gibson et al. [25] evaluated accelerometer data with wavelet transforms and principal component analysis to detect falls and utilise compressive sensing based techniques to reduce transmission information. They further utilise multiple classifiers with a majority voting system to improve performance over a single classifier for robust classification [24]. Sukor et al. [16] used accelerometer data from the MobiFall dataset [26] for signal processing and features selection. A number of time-domain and frequency domain features were used including Spectral Density and Spectral Energy. Kwolek et al. [8] applied support vector machines with accelerometer signals and image evaluation for fall detection. Hsieh et al. [13] presented the hierarchical fall event algorithm that uses a dual threshold and machine learning based approach for detection of falls from triaxial accelerometer with sensitivity, specificity and accuracy values above 98%. Zhong et al. [17] presented a real-time algorithm system based on the thresholds of velocity and displacement to classify falls. A second order filter was applied to the signal to minimize the impact of drift on vertical velocity.

Current work on chaotic and fractal analysis of human movements is limited to gait analysis and human walk. Human gait models have been determined to possess elements of chaotic systems in [27]. A local dynamic stability analysis of walking activity and human gait has determined a positive Lyapunov Exponent (LE) [28,29,30,31]. A positive LE is a signature characteristic of chaotic systems and a measure of sensitivity to small perturbations. Recently, [28,32] have associated local dynamic stability with the risk of falls. Morbidoni et al. [33] utilise electromyography signals for deep learning classification of stance, swing phases during natural walking. Pairot et al. [34] determined the validity of commercially available, wearable sensors by monitoring gait during running and concluded that only few metrics measured by commercially available sensors are valid. Park et al. [35] developed a real-time healthcare monitoring system for monitoring gait and vital signs, and utilised machine learning classification for health conditions, such as disordered gait and onset of stroke. Margiotta et al. [36] utilised a wearable device wireless system to analyse time gait variability, for early diagnosis of health conditions. Nguyen et al. [37] captured gait characteristics with multiple wearable body sensors and performed gait classification to determine subject groups with abnormalities. Schneider et al. [38] proposed a gait analysis system to determine gait parameters and speed. The authors utilised a camera and an accelerometer sensor for discriminating various gait speeds through frequency domain gait features. Coviello et al. [39] utilised embedded devices with inertial sensors to propose a large multi-sensor platform for measurement of activities through reduced single node complexity and guaranteeing time synchronization for acquired samples. Sahoo et al. [40] proposed an early detection technique for inertial sensor based system to detect gait events early and reduce the effect of delay in powered prosthetic and assistive devices. Apart from stability analysis, fractal dynamics of walk and human gait has also been analysed in [41,42,43]. Fractal dimensions have been used in biomedical systems for detection of anomalies [44,45]. Koutsiana et al. [44] evaluated fractal dimensions on wavelet transformed data for detection of fetal heart sounds, while Zhang et al. [45] used fractal dimensions to detect brain anomalies. current work has not investigated fractal features for training machine learning algorithms for fall and activities, let alone an embedded reconfigurable device implementation.

Recent published work with FPGAs and SoCs with programmable logic resources such as the Xilinx Zynq system either do not provide power consumption analysis or suffer from lower accuracies with accelerometer sensors for fall detection. Vision-based approaches have limitations, while higher accuracy is also achieved at the cost of higher power consumption. Senouci et al. [46] utilised spatio-temporal information from camera images including wavelet transform, object bounding box height, width, aspect ratio and motion variation with hardware acceleration on a Zynq device to classify falls in real-time with Support Vector Machine (SVM) and Adaboost algorithms. However, their implementation did not give any power consumption values. Ali et al. [47] implemented a sensor-based FDS on a Zynq System on Chip (SoC) device which applies DWT and PCA for feature extraction and a binary decision tree for classification. The system suffered from lower accuracy and no power consumption analysis has been performed. Ong et al. in [48,49] presented an FPGA-based architecture for visual fall detection with a CMOS camera. They achieved a frame rate of 60 fps for VGA resolutions of 640 × 480 with a pixel processing pipeline which implements feature extraction. Furthermore, the design is optimised in [49] for power giving a reduction of up to 33% in power consumption from their initial design. The technique not only suffered from the limitations of a vision-based systems, but resulted in a high power consumption of 5.2 W which is not feasible for an embedded wearable device. Abdelhedi et al. [50] proposed an FPGA based solution for fall detection on a Zybo board with a single accelerometer. Their work used threshold method with the sum-vector of three accelerometer axes and the body tilt angle for fall detection. All the processing is performed in software on the ARM core, while only the detection decision is sent to the programmable logic for fall detection indication (blinking LEDs). The work was further expanded in [51] by implementing a hardware core for the above mentioned operations in programmable logic on a Zybo FPGA board. To the best of our knowledge, this is the first implementation of a fractal feature accelerator using multi-level DWT for classification of falls from ADL.

## 3. Fall Detection System Overview

**Figure 1:**
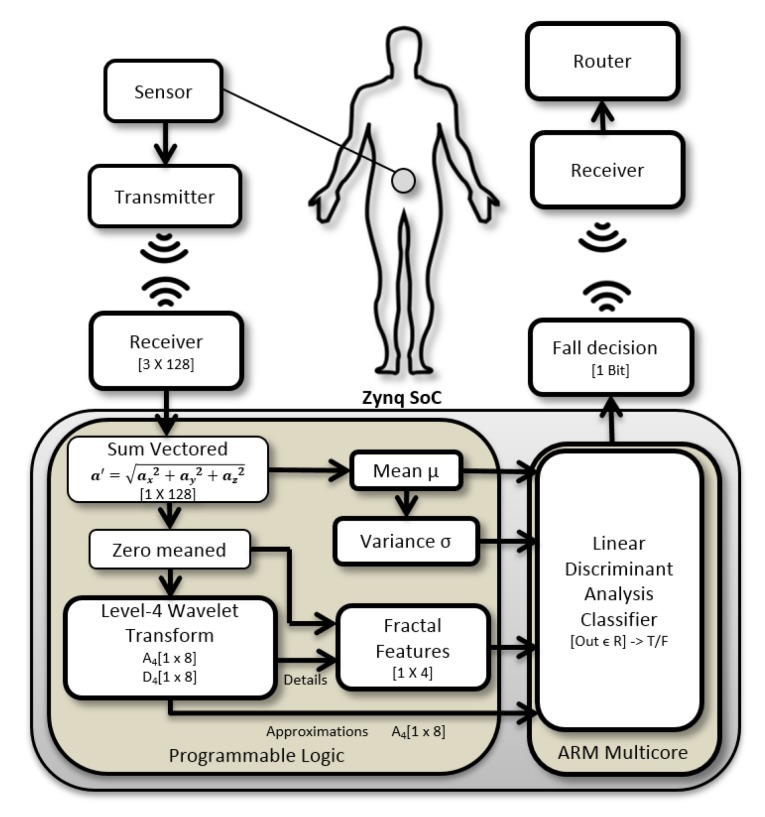
Wearable fall detection system overview.

The proposed fall detection system concept is illustrated in Figure 1 and is designed to evaluate wearable accelerometer sensors with efficient machine learning algorithms implemented on an embedded reconfigurable Zynq device.

While the proposed Zynq SoC design is potentially a prime candidate for a wearable/body mounted configuration, in this work, the proposed system is designed as a Zynq device base station for portability, sustainability and scalability, and performs ADL/fall classification and detection derived from dataset signals.The fall classification and detection decision is transmitted from the Zynq SoC device to the wireless router, from where a medical aid centre is notified for an immediate medical response.

The Zynq SoC device is a development board and consists of dual-core ARM processor and a Programmable Logic (PL) core. The Zynq device has 6.3 in × 6.3 in dimensions with a SoC measuring 19 mm × 19 mm. The Zynq board includes 256 KB on chip RAM, 512 MB board RAM, Serial Peripheral Interface (SPI) and Inter-Integrated Circuit (I2C) interfaces and 12-bit Analog to Digital Converters (ADC). The system has on chip voltage and temperature sensors. External accelerometer sensors can be connected wirelessly to the board or directly through the ADC and communicate directly with the PL. The device works with 12 V battery operated or external power converter. The movement signals can be obtained from any wearable accelerometer sensor in units of G-force (g), with similar specifications to the dataset by Kwolek et al. [8], against which the design is validated. The proposed Zynq SoC design is compatible with accelerometer sensors, such as x-IMU [52] and ADXL345-EP [53] which have a programmable sampling rate of 32 Hz and a full scale range of −8 to +8 g at 12-bit resolution. Window segments of 128 samples from each of the tri axis acceleration signals $a_{x},a_{y}$ and $a_{z}$ along three axes of motion x,y and *z* are processed to obtain the sum vectored signal $a^{\prime }=\sqrt{a_{x}^{2}+a_{y}^{2}+a_{z}^{2}}$. The process captures the signal variations along the three axis of motion into a single signal and reduces the processing time. The mean $\mu_{a^{\prime }}$ of the sum vectored signal $a^{\prime }=\left\{a^{\prime }\left(n\right)\right\}$ where n={1,…,128}, is computed and the sum vectored signal is zero meaned, $a=a^{\prime }-\mu_{a^{\prime }}$. The zero mean signal a={a(n)} for n={1,…,128} is then wavelet transformed with a Daubechies-4 DWT function to obtain the DWT approximation $A_{1}$ and detail coefficients $D_{1}$ at level 1. The process is repeated 4 times to achieve a 4-level wavelet transform with a DWT output signal of size [1×8] for each of the approximation and detail coefficients. The variance of the DWT detail coefficients $D_{i}$ and the variance $\sigma_{a}^{2}=\sum_{n=1}^{N}{\left(a\left(n\right)-\mu_{a}\right)}^{2}/\left(N-1\right)$ of the zero meaned, sum vectored accelerometer signal a with mean $\mu_{a}=0$ and N=128 are used for fractal analysis, since the variance of detail coefficients can be utilised to determine fractal dimensions according to Equations (25)–(27). The fractal dimensions along with the mean $\mu_{a^{\prime }}$ and variance $\sigma_{a^{\prime }}^{2}$ of the sum vectored accelerometer signal $a^{\prime }=\left\{a^{\prime }\left(n\right)\right\}$ for n={1,…,128} and DWT approximation coefficients $A_{4}$ at level 4 are used as a feature set for classification and detection of falls. The classification is carried out on the feature set with machine learning classifiers on the ARM core of a reconfigurable embedded system, as shown in Figure 1. Multiple DWT level analysis is a computationally intensive process and requires hardware implementation to investigate resource usage with associated DWT levels and fall detection accuracy. The fractal dimensions are calculated from the wavelet coefficients on the reconfigurable logic hardware accelerator.

**Figure 2:**
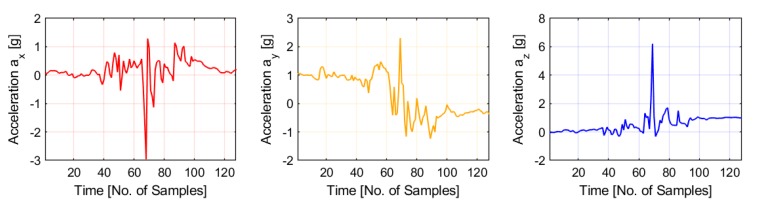
Three axes accelerometer signals $a_{x}$, $a_{y}$ and $a_{z}$ for falls.

*Dataset:* The proposed DWT based fractal feature and machine learning algorithms for FDS were trained, tested and validated with Matlab and publicly available fall accelerometer data by Kwolek et al. [8]. The data consists of falls and various activities of daily life including walking, kneeling, picking up objects, standing up, sitting down and lying. The data is obtained from an Inertial Measurement Unit with 12 bit three axis accelerometer and 16 bit gyroscope. The device was worn with the pelvis by 5 individuals who performed different kind of falls, including backward, forward and lateral falls. The data obtained from sensors is transmitted wirelessly from the device through Bluetooth. The sampling rate of the accelerometer is 32 Hz (overall sampling rate is 256 Hz for multichannel sensors). In our work, we utilise the accelerometer data for testing proposed features and algorithm. The accelerometer sensor values vary from −8 to +8 g. Three axes of accelerometer values are shown in Figure 2 with 128 sample segments for fall activity. Samples from each of the activities were used for training and validation of the proposed architecture. The input accelerometer data and the target outputs are stored as C arrays in the Xilinx Software Development Kit (SDK). The arrays are loaded into the Zynq memory and the accelerometer values are read in a loop. The data values are used as inputs into the hardware accelerator and the output features obtained from the hardware accelerator are used for LDA classification algorithm executed on the ARM core. The classification results obtained are then compared with the target outputs in the array and classification accuracy is calculated.

While the classification performance values have been computed for the given dataset. The fractal dimension, according to Mandelbrot [4] has a positive correlation with irregularity of a signal, and can be used as a measure of signal irregularity. The different irregularity characteristics of fall signals is a generic observation, which distinguishes all fall signals composed of a spike with ADL. The ADL signals are mostly composed of irregular episodic variations and hence can be distinguished from falls based on their irregularity characteristics.

## 4. Signal Processing and Fractal Analysis

### 4.1. Discrete Wavelet Transform

The Discrete Wavelet Transform is a projection of the sum vectored and zero mean accelerometer signal a={a(n)} where n={1,…,N} and N=128 samples, on a family of basis functions $\phi_{i,k}\left(n\right)$ and $\psi_{i,k}\left(n\right)$. The family of basis functions is obtained from the translations and dilations of the scaling function ϕ(n) and mother wavelet ψ(n), which are defined as:

(1) $$\begin{array}{cc} \phi_{i,k}\left(n\right) & =2^{-\frac{i}{2}}\phi \left(2^{-i}n-k\right) \end{array}$$

(2) $$\begin{array}{cc} \psi_{i,k}\left(n\right) & =2^{-\frac{i}{2}}\psi \left(2^{-i}n-k\right) \end{array}$$

where *k* represents discrete translations and $2^{i}$ are dyadic dilations. The DWT on signal a(n) can be given as:

(3) $$\begin{array}{cc} A_{i}\left(k\right) & =\sum_{n}a\left(n\right)\phi_{i,k}\left(n\right) \end{array}$$

(4) $$\begin{array}{cc} D_{i}\left(k\right) & =\sum_{n}a\left(n\right)\psi_{i,k}\left(n\right) \end{array}$$

where $A_{i}$ and $D_{i}$ are wavelet approximation and detail coefficients for the sum vectored and zero mean accelerometer signal a(n), respectively at level *i*. Index k of wavelet coefficients corresponds to the shift index of the scaling and mother wavelet functions. The approximations also known as low pass wavelet coefficients $A_{i}$ are used as an input signal in Equations (3) and (4) multiple times to produce 4-level DWT approximations $A_{4}$ and details $D_{4}$. The final approximations $A_{4}$ and details $D_{4}$ are a vector of size [1 × 8 ] each, as shown in FDS overview in Figure 1. The 4-level DWT approximations $A_{4}$ are directly used as features for classification in the FDS system, while the $D_{4}$ details are used to compute fractal dimension features for the signal as illustrated in Figure 1. The use of fractal features in the fall classification model is based on a fractal analysis of falls and activities, which show that fractal dimension can be used as an irregularity metric of the signal to discriminate between falls and activities. Section 4.2 and Section 4.3 discuss stationarity tests and the fractal analysis of falls and activities, respectively.

### 4.2. Stationarity Tests

While there are many fractal analysis methods, such as Detrending Fluctuation Analysis (DFA) and Power Spectral Density (PSD), the ARFIMA method has been demonstrated to be superior to other methods for complex processes [6]. AFRIMA, however requires a process to be stationary or converted to a stationary process for analysis. Therefore stationarity testing of falls and ADLs signals or conversion to a stationary process in case of non-stationarity is imperative for computation of fractal parameters. The stationarity and non-stationarity test are discussed below, and a fractal analysis with ARFIMA is presented in next section. The ADF and KPSS tests are used for stationarity testing. ADF assumes the signal is non-stationary and tests for the presence of unit root. The test for unit root [54] determines if the process is non-stationary with stationary increments, i.e., first order difference stationary. The sum vectored, zero mean signal a={a(n)} can be viewed as a unit root process with stationary increments in an ADF test. While KPSS test assumes that the signal is level stationary and tests whether the assumption is correct or not, which results in a double validation strategy.

#### 4.2.1. Augmented Dickey-Fuller Test

The Augmented Dickey-Fuller (ADF) test [54] determines the stationarity of the process, which represents the human body movements responsible for signal variations. Different types of ADF tests are available. The form evaluated here is the ADF constant stationary with no trend, since signal values in our case do not exhibit long term time trends. However, the non-stationarity of signals against trend stationarity are also confirmed with the KPSS test in the next Section 4.2.2 for rigorous treatment. ADF test provides a rejection decision for a unit root process hypothesis $H_{0}$ of the form given in (5) against an alternative hypothesis $H_{1}$ in (6):

(5) $$\begin{array}{cc} H_{0}:\;a(n) & =a(n-1)+\sigma_{1}\Delta a(n-1)+\cdots +e\left(n\right) \end{array}$$

(6) $$\begin{array}{cc} H_{1}:\;a(n) & =\gamma a(n-1)+\sigma_{1}\Delta a(n-1)+\cdots +e\left(n\right) \end{array}$$

where,

- Δ is the difference operator with Δa(n)=a(n)−a(n−1).
- e(n) is an innovation process.
- γ is an autoregressive coefficient with value <1.

The test gives a value of 0 or 1. A value of 0 means failure to reject the null hypothesis, while 1 means rejection of the null hypothesis. A *p*-value of less than 0.05 (5%) is used for the rejection decision and represents 95% confidence level for accepting the alternative hypothesis of stationarity. The hypothesis are as follows:

Null Hypothesis $H_{0}$: γ=1 indicated by a test output of 0 means failure to reject the null hypothesis of a unit root process. The accelerometer signal generating process is considered to be non-stationary.

Alternative Hypothesis $H_{1}$: γ<1 indicated by a test output of 1 means rejection of a unit root process in favor of alternative model. The accelerometer signal generating process is considered to be stationary. The Shwert rule for determining maximum lags for the ADF test [55] is given as:

(7) $$\begin{array}{c} p_{max,ADF}=\left[12\times {\left(\frac{N}{100}\right)}^{1/4}\right] \end{array}$$

where *N* is the total number of samples. The tests were performed with lag values according to two criteria. Firstly, the ADF test was performed with the maximum lag value $p_{max}$. Ng et al. [56] suggested a lag length selection procedure based on first selecting the maximum lag value $p_{max}$ for p. If the *t*-statistic representing the significance is greater than 1.6 then $p_{max}$ is used as the final lag value for the test, otherwise the lag was reduced by one and the process repeated. Secondly, the lag values were obtained from Akaike Information Criterion (AIC) [57] and used for ADF tests. Different criteria may provide different lag values for the test, since there is no single criteria which may apply to all cases, we tested stationarity against both lag selection criterias, which confirmed same results for the stationarity/non-stationarity tests.

#### 4.2.2. Kwiatkowski-Phillips-Schmidt-Shin Test

Kwiatkowski-Phillips-Schmidt-Shin (KPSS) test assumes a signal is trend stationary and tests for non-stationarity. The null hypothesis in KPSS test that the accelerometer signal generating process is stationary is opposite to the ADF test. The null hypothesis $H_{0}$ is given in Equation (8) representing a stationary process. While, the alternative hypothesis of unit root $H_{1}$ is given in Equation (10).

(8) $$\begin{array}{cc} H_{0}:\;a(n) & =c(n)+\lambda n+v_{1}\left(n\right),\;\mathrm{where} \end{array}$$

(9) $$\begin{array}{cc} c(n) & =c(n-1)+v_{2}\left(n\right) \end{array}$$

(10) $$\begin{array}{cc} H_{1}:\;a(n) & =a(n-1)+d_{c}+\lambda n+e\left(n\right) \end{array}$$

where,

- λn represents a deterministic trend with coefficient λ and the number of samples *n*.
- $d_{c}$ represents drift constant.
- c(0) for n=0 is fixed and represents an intercept. The subsequent values are calculated from (9).
- e(n) is an innovation process.
- $v_{1}\left(n\right)$ represents a stationary process.
- $v_{2}\left(n\right)$ is a distributed process which is identically distributed and independent with 0 mean.

The test gives a value of 0 or 1. A value of 0 means failure to reject the null hypothesis, while 1 means its rejection. A *p*-value of less than 0.05 (5%) is used for the rejection decision and represents 95% confidence level for accepting the alternative hypothesis of non-stationarity. The hypothesis are as follows:

Null Hypothesis $H_{0}$: indicated by a test output of 0 means failure to reject the null hypothesis of a stationary process. The signal generating process is therefore considered to be stationary.

Alternative Hypothesis $H_{1}$: indicated by a test output of 1 means rejection of the null hypothesis in favor of the alternative model. The signal generating process is considered to be non-stationary. The rule for determining maximum lags is given by Kwiatkowski et al. [58] as:

(11) $$p_{max,KPSS}=\sqrt{N}$$

The tests were performed with fewer lags and the sensitivity was measured by adding more lags.

#### 4.2.3. Stationarity Results

The results of both the ADF and KPSS tests show that both the fall and ADL signals are non-stationary, which is indicated by positive results for the ADF tests and inability to reject the null hypothesis. While, the KPSS tests were negative and rejected the null hypothesis with a high confidence value of more than 95%, corresponding to a *p*-value of less than 0.05. Furthermore, applying first order difference filter results in stationary signals, as supported by the results of ADF test which rejects the null hypothesis with confidence of greater than 95% and a *p*-value of less than 0.05. For ARFIMA based fractal analysis in Section 4.3.1, which requires conversion to a stationary signal, the first difference filter operation is considered sufficient for conversion to stationary signal and is used for computing fractional difference parameter *d*, related to the Hurst exponent and fractal dimensions.

### 4.3. Fractal Analysis: ARFIMA

The fractal parameter analysis ARFIMA is based on the modelling for better accuracy of results. In [59], Box et al. presented a family of Autoregressive Integrated Moving Average (ARIMA) models to introduce short-term relationships in time varying processes. ARFIMA models are a generalization of ARIMA models based on fractional calculus. ARFIMA models can be used to find fractal dimensions and provide a fractional value of the differencing parameter *d*, which is directly related to the Hurst exponent *H*. The fractional value of *d* is bounded by [−0.5,0.5] and applies only to a stationary process. ARFIMA can be applied to a non-stationary signal a(n) by initially converting to a stationary process through the difference operator, s(n)=a(n)−a(n−1). In contrast, the fractional parameter $\tilde{d}$ for the non-stationary process a(n) can then be obtained by adding 1 to the fractional parameter *d* for the stationary process s(n) [7]. ARIMA models have three components: Autoregressive AR(r), Moving Average MA(q) and the Integrated part I(d). The autoregressive AR(r) term describes the value s(n) by a weighted-sum of previous *r* values and a random variable ϵ(n):

(12) $$s\left(n\right)=\alpha +\sum_{l=1}^{r}\zeta_{l}s\left(n-l\right)+\epsilon \left(n\right)$$

where $\zeta_{1},\zeta_{2},\cdots ,\zeta_{r}$ are autoregressive coefficients and α is a constant. The autoregressive terms decay over time for stationary processes. The MA(q) term describes the current value s(n) by a weighted-sum of previous *q* random perturbations ϵ(n−1),⋯,ϵ(n−l).

(13) $$s\left(n\right)=\mu_{s}+\sum_{l=1}^{q}\theta_{l}\epsilon \left(n-l\right)+\epsilon \left(n\right)$$

where $\theta_{1},\theta_{2},\cdots ,\theta_{p}$ are moving average coefficients and $\mu_{s}$ is the mean of s(n). The integrated I(d) part represents the order of difference *d* required for the ARIMA Model. It specifies whether the observed values are directly modelled; d=0 or their differences d=1,2,⋯ are modelled. Given a lag operator Ls(n)=s(n−1) for all n>1, where $L^{l}s\left(n\right)=s\left(n-l\right)$.

(14) $$\begin{array}{cc} \Delta s(n) & =s(n)-s(n-1)\\ \; & =(1-L)s(n) \end{array}$$

(15) $$\begin{array}{cc} \Delta^{d}s\left(n\right) & ={\left(1-L\right)}^{d}s\left(n\right) \end{array}$$

The ARIMA model can be described as:

(16) $$\begin{array}{cc} \left(1-\sum_{l=1}^{r}\zeta_{l}L^{l}\right){\left(1-L\right)}^{d}\left(s\left(n\right)-\mu_{s}\right) & =\left(1+\sum_{l=1}^{q}\theta_{l}L^{l}\right)\epsilon \left(n\right) \end{array}$$

In ARIMA models *d* is an integer, while in fractional ARFIMA(r,d,q) models capture fractal dynamics with real values for *d*. An ARFIMA(r,d,q) model for the accelerometer difference signal s(n) can be described by Equation (16), where the fractional difference operator $\Delta^{d}={\left(1-L\right)}^{d}$ can be represented by a binomial expansion for real number *d* with Gamma function as:

(17) (1−L)d=∑l=0∞dl(−L)l=∑l=0∞Γ(d+1)Γ(l+1)Γ(d+1−l)(−L)l

with general form of ARFIMA(r,d,q) process defined as:

(18) $$\Theta \left(L\right){\left(1-L\right)}^{d}s\left(n\right)=\Theta \left(L\right)\epsilon \left(n\right)$$

where *d* is between −0.5 to 0.5 and represents the self-similarity of the ARFIMA process. For non-stationary process a(n), the fractional difference value $\tilde{d}$, signal spectral exponent β, Hurst exponent *H* and fractal dimension fd can then be calculated from *d* as:

(19) $$\begin{array}{cc} \tilde{d} & =d+1 \end{array}$$

(20) $$\begin{array}{cc} \beta & =2\tilde{d} \end{array}$$

(21) $$\begin{array}{cc} H & =\frac{\beta -1}{2} \end{array}$$

(22) $$\begin{array}{cc} fd & =2-H \end{array}$$

#### 4.3.1. Fractal Dimensions of Falls with ARFIMA

**Figure 3:**
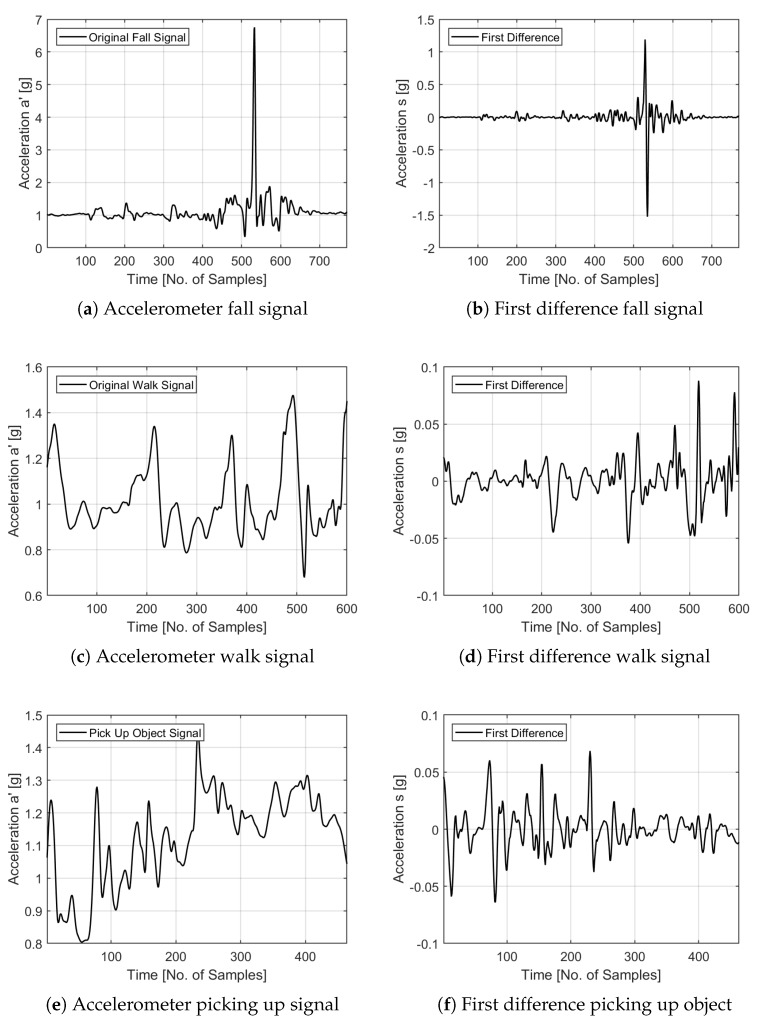
Original and first difference signals.

Estimation of fractal dimensions was performed after application of the first order difference filter to the activity signals to convert the non-stationary process into a stationary process for ARFIMA modelling. We fitted 20 ARFIMA (r,d,q) models with r∈[0,4] and q∈[0,4] to the output of the first difference filter and retained fractional difference *d* with the lowest log likelihood and AIC values for accuracy and parsimony along with the model fitness values. For illustration purposes, the original signals for fall, walking and picking up objects along with their first difference signals are shown in Figure 3. While, the selected ARFIMA models for falls, walking and picking up objects with the lowest log likelihood and AIC values are illustrated in Table 1. The fractional difference *d* values of the selected ARFIMA models with best goodness of fit statistics are then utilised for computing fractional integration coefficient $\tilde{d}$ for the original non-stationary process and the corresponding fractal dimension using Equations (19)–(22). The mean values for fractional integration coefficient $\tilde{d}$, Hurst parameter *H* and fractal dimensions fd are given in Table 2 for all the falls and ADLs. The value of Hurst exponent H=0.49 and fractal dimension 1.01 for falls demonstrates a clear distinction in irregularity characteristics of falls compared to ADLs. ADLs show higher irregularity w.r.t. the dimension parameter. It is clear from analysis that the value of fractal dimension for falls at 1.01 can act as a good discriminant for fall classification, since all other activities have relatively higher values. The fractal dimension was not calculated for “lying” in Table 2 because of an unreliably high Standard Deviation (SD) greater then the mean value, which results in a fractal dimension of 2.

While, ARFIMA is more rigorous and robust for computation of fractal parameters for complex processes [5,6]. The use of stationary signals, higher computations and selection of ARFIMA models are not feasible for an embedded approach, where signals are non-stationary or stationarity characteristics of the process are not known. DWT based computation of fractal dimensions does not suffer from the limitations of ARFIMA and can be directly applied to non-stationary signals for real-time computation of fractal dimensions.

## 5. Proposed Fall Classification with Fractal Features

This section discusses the proposed fall classification algorithm based on fractal features for an embedded wearable device. The previous Section 4.3.1 utilises ARFIMA analysis to establish that fractal dimension for falls is distinct with a mean value of 1.01, as compared to other activities which have a mean fractal dimension of at least 1.5, as illustrated in Table 2. While, ARFIMA analysis is robust and less prone to error for complex processes [5,6]. ARFIMA is computationally expensive and requires computing various ARFIMA models for a number of different parameters before a selection of the best-fit could be made based on goodness of fit statistic functions. Hence fractal dimension computations based on ARFIMA analysis are not suitable for an embedded system to compute and utilise fractal features for detection of falls in a low latency wearable device with real time constraints. We show that such a system can utilise DWT based method for computation of fractal dimensions for non-stationary falls and activities signals in an embedded system with low latency requirements. The proposed reconfigurable embedded FDS therefore utilises DWT for computation of fractal features. The mathematical basis for the DWT based method are given in next Section 5.1 and the classification algorithm used in the FDS is discussed in Section 5.2. While, classification accuracy improvements with fractal features are presented in Section 5.3.

### 5.1. DWT Based Fractal Features

The fractal dimensions can be computed from the Hurst exponent *H*, which can be calculated for non-stationary accelerometer signals by use of DWT detail coefficients [9,10]. In the DWT method, high pass wavelet decomposition filter has a frequency band of $f_{s}/2^{i+1}<\omega <f_{s}/2^{i}$ for the *i*th level wavelet detail coefficients $D_{i}$ for a sampling frequency of $f_{s}$. The variance of the detail coefficients can therefore be used as the power spectrum density S(ω) of the original signal. The power spectrum density of the signal can be given in terms of variance $\sigma_{a}^{2}$ of the non-stationary, sum vectored and zero mean signal a(n) and the power spectrum exponent β, as:

(23) $$S\left(\omega \right)=\frac{\sigma_{a}^{2}}{{∥\omega ∥}^{\beta }}$$

Replacing the above with variance of the detail coefficients results in the following equation:

(24) $$var\left(D_{i}\right)=\frac{\sigma_{a}^{2}}{{\left(2^{i}\right)}^{\beta }}$$

The power spectrum exponent β can be calculated from the above equation as:

(25) $$\beta =\frac{log_{2}\left[\sigma_{a}^{2}/var\left(D_{i}\right)\right]}{log_{2}\left[2^{i}\right]}$$

The Hurst exponent *H* and fractal dimension fd can be calculated as:

(26) $$\begin{array}{c} H=\frac{\beta -1}{2} \end{array}$$

(27) $$\begin{array}{c} fd=2-H \end{array}$$

### 5.2. Proposed Algorithm

**Figure 4:**
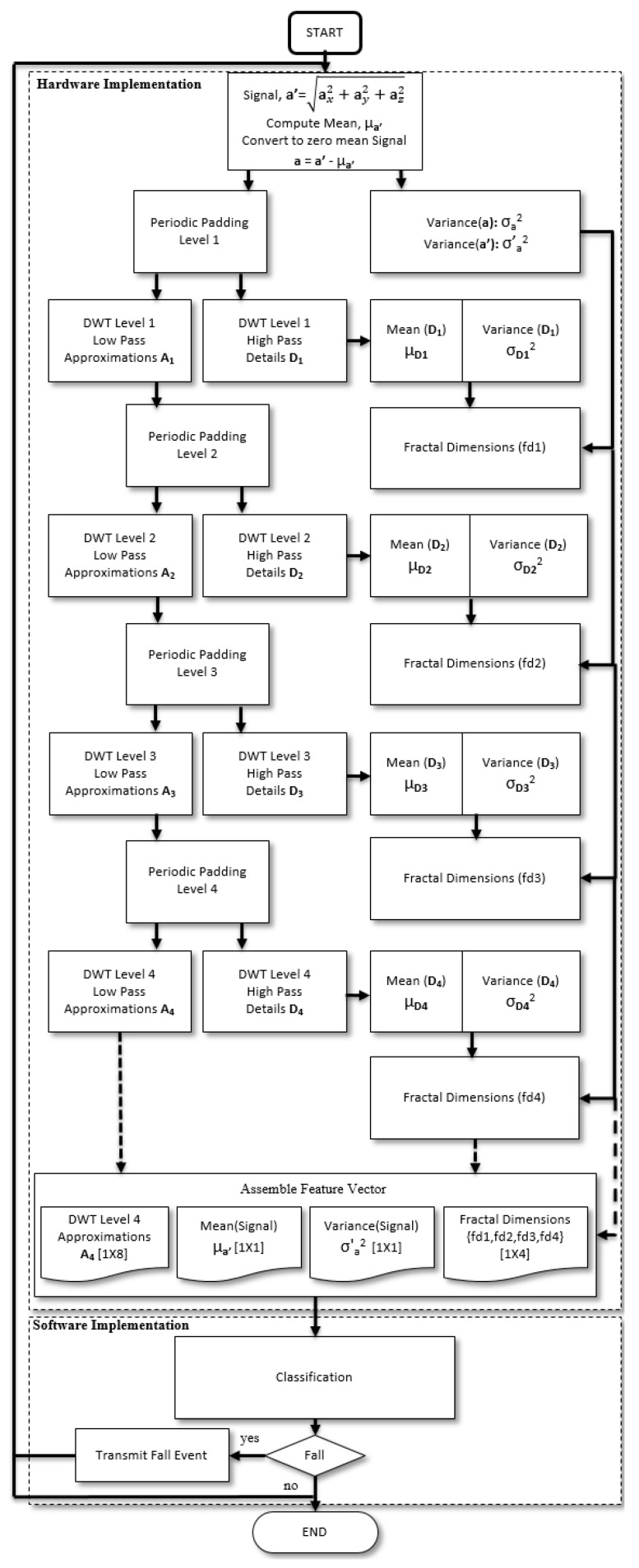
Flow chart of the proposed scheme.

The proposed algorithm performs fall detection and classification from ADLs based on features obtained from multi-wavelet transform, mean of the signal, variance of the signal and the subsequent computation of fractal dimensions at each level of the multi-level wavelet transform. The feature extraction part is performed in hardware, where the classification is done on the ARM core in software. The steps of the proposed algorithm are given below and the flow chart is illustrated in Figure 4 with hardware and software implemented components. The following steps are performed on 128 sample windows of accelerometer readings with 50% overlapping between the windows for each of the three accelerometer axis.

1. Compute the sum vectored signal $a^{\prime }$ using, $a^{\prime }=\sqrt{a_{x}^{2}+a_{y}^{2}+a_{z}^{2}}$ of the tri axis accelerometer signals, $a_{x}$, $a_{y}$ and $a_{z}$.
2. Compute the mean $\mu_{a^{\prime }}$ of the sum vectored signal $a^{\prime }$ and convert to a zero mean signal, $a=a^{\prime }-\mu_{a^{\prime }}$.
3. Compute variance $\sigma_{a^{\prime }}^{2}$ of the sum vectored signal $a^{\prime }$ to use as a feature.
4. Compute variance $\sigma_{a}^{2}$ of the sum vectored and zero mean signal a for computation of fractal dimensions.
5. Perform Periodic padding of the zero mean signal a and compute first-level wavelet transform approximations $A_{1}$ and details $D_{1}$.
6. Compute the mean $\mu_{D_{1}}$ and variance $\sigma_{D_{1}}^{2}$ of the detail coefficients and use the variance $\sigma_{a}^{2}$ of the signal a in step 4 to compute the fractal dimension fd1 at level 1.
7. Perform Periodic padding of the first-level wavelet detail coefficients and compute second-level wavelet transform approximations $A_{2}$ and details $D_{2}$.
8. Compute the mean $\mu_{D_{2}}$ and variance $\sigma_{D_{2}}^{2}$ of the second level detail coefficients and use the variance $\sigma_{a}^{2}$ of the signal a in step 4 to compute the fractal dimension fd2 at level 2.
9. Perform Periodic padding of the second-level wavelet detail coefficients and compute third-level wavelet transform approximations $A_{3}$ and details $D_{3}$.
10. Compute the mean $\mu_{D_{3}}$ and variance $\sigma_{D_{3}}^{2}$ of the third level detail coefficients and use the variance $\sigma_{a}^{2}$ of the signal a in step 4 to compute the fractal dimension fd3 at level 3.
11. Perform Periodic padding of the third-level wavelet detail coefficients and compute fourth-level wavelet transform approximations $A_{4}$ and details $D_{4}$.
12. Compute the mean $\mu_{D_{4}}$ and variance $\sigma_{D_{4}}^{2}$ of the fourth level detail coefficients and use the variance $\sigma_{a}^{2}$ of the signal a in step 4 to compute the fractal dimension fd4 at level 4.
13. Assemble a feature vector of wavelet approximations at level 4, $A_{4}$ [1×8], mean of the sum vectored signal, $\mu_{a^{\prime }}$ [1×1], variance of the sum vectored signal, $\sigma_{a^{\prime }}^{2}$ [1×1] and instantaneous fractal dimensions, {fd1, fd2, fd3, fd4} of dimensions [1×4] at all four levels of wavelet transform.
14. Perform classification with LDA machine learning algorithm between falls and no falls.
15. In case of a fall, transmit fall event occurrence for medical aid response.
16. Repeat from step 1 for the next 128 sample window with 50% overlap.

### 5.3. Classification Performance of Fractal Features

The classification performance of the DWT based fractal features was determined by implementing the feature extraction process and the LDA machine learning algorithm in Matlab. The algorithm was tested and evaluated on a public fall dataset by Kwolek et al. [8]. The fall dataset [8] is discussed previously in Section 3. The classification performance for different set of features and the proposed scheme are evaluated in Table 3. LDA classification algorithm finds the maximum separation between falls and other activities by maximising between the class variance and minimising the variance within the classes. The fractal features alone can provide high performance with significant classification accuracy of 91.84% with LDA classifier, as compared to level 2, 3 and 4 wavelet coefficients as illustrated in Table 3. In addition, combining fractal and wavelet coefficients results in higher accuracy of 95.61%. Classification accuracy further increases to 99.38% for the proposed scheme, when mean and SD of the signals are added to the feature set, as illustrated in Table 3.

Table 4 compares the performance of the proposed system features and classifier with state-of-the-art work. The latest works on FDS have utilised a number of machine and deep learning classifiers including SVM, Quadrature SVM (QSVM), Random Forest (RF), K-Nearest Neighbour (KNN), Decision Tree (DT), Ensemble Bagged Tree (EBT), Artificial Neural Network (ANN) and deep hybrid Random Neural Network (RNN) as illustrated in Table 4. The systems presented in [60,61,62,63] use a large number of features as compared to our system, which utilises only four features namely, fractal dimensions, DWT level 4 coefficients, mean and SD. The works in [60,62,63] leverage additional gyroscopic sensors apart from the tri-axes accelerometer, while [61] uses multiple accelerometer sensors attached to waist, chest and thigh. Our proposed system utilises only one accelerometer sensor attached to the pelvis to achieve the best performance results. The systems in [60,62,63] utilise ensemble learning techniques to achieve their best results along with other classifiers, such as SVM, QSVM and KNN. The work in [64] utilises raw accelerometer values with a deep hybrid RNN classifier, however suffers from the complexity of a deep neural network and provides 7.1% less accuracy than our proposed scheme. The proposed system utilises the LDA classifier and fractal features to give the best performance results in terms of accuracy, sensitivity and specificity of 99.10%, 99.90% and 99.38%, respectively, while utilising low number of features.

## 6. Proposed Algorithm Performance Analysis

The software performance of the proposed algorithm with DWT based fractal feature extraction and LDA classification algorithm was determined on an embedded ARM cortex A9 at 666 MHz with C/C++ implementation. Xilinx SDK was used for running and evaluating embedded code.

**Figure 5:**
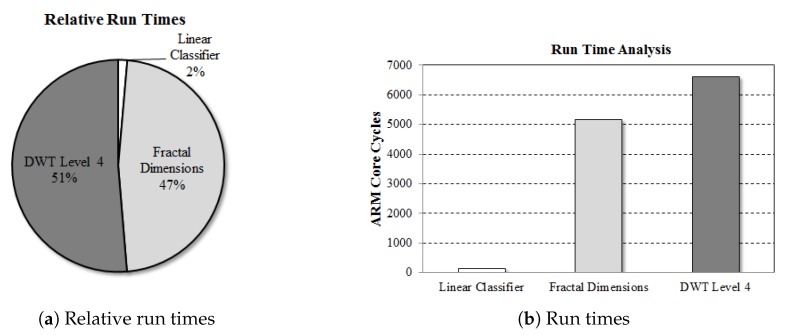
Run time analysis of the proposed algorithm.

The multi-level wavelet transform and fractal dimension algorithms were observed to be more computationally intensive than the LDA classification algorithm. The DWT algorithm consumed the highest percentage of the total runtime at 51%, slightly higher than the fractal computations which were also computationally expensive at 47% of the total runtime cost. The higher computational load of DWT algorithm resulted from four convolution operations performed for each of the four levels of wavelet transform. Overall, the four convolutions consume more cycles compared to the entire algorithm with approximately 6700 cycles of the embedded ARM core, where the first convolution is computed for 128 samples, second for 64, third for 32 and fourth for 16. The fractal algorithm computes means and variances from the obtained DWT coefficients at each level and utilises the signal variances to compute fractal dimensions, which is also computationally expensive. The fractal algorithm consumes approximately 5000 cycles of the embedded ARM core. The computationally intensive part of the fractal algorithm is the computation of variances of the wavelet detail coefficients obtained from the four convolutions, where variance computations involve subtraction of each accelerometer sample with the mean of the signal and calculation of the square of each term, corresponding to product of each term with itself. The overall computational complexity of the algorithm is $O(n^{2})$, which is due to the variance computations performed in the algorithm. Moreover, the machine learning classification performed with the LDA algorithm consumed only 2% of the total runtime cost, which was not considered significant enough for hardware acceleration. The relative execution times are illustrated in Figure 5a, while the run times of the proposed algorithm are shown in Figure 5b.

Due to significantly higher computational requirements, fractal computations and multi-level wavelet transforms were accelerated with custom designed reconfigurable hardware architecture to achieve higher dividends for power, latency and performance per Watt metrics. While LDA classification algorithm was executed as embedded software program on the ARM processor.

## 7. FDS Hardware/Software Co-Design

**Figure 6:**
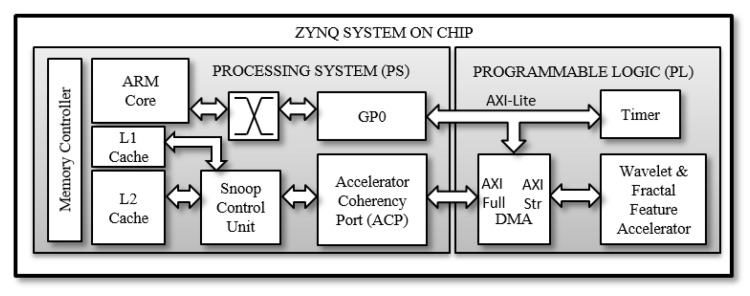
Zynq SoC design for a fall detection system.

The hardware/software co-design along with the hardware architecture, design and optimizations are discussed in this section. The design flow consists of synthesizing the high level C++ code with the Xilinx Vivado high level synthesizer to an equivalent Register Transfer Language (RTL). The RTL is then exported in the form of a Vivado Intellectual Property (IP). The IP along with other peripherals and the ARM core IP block are imported in the Vivado block diagram environment. The system is synthesised and exported to the Xilinx SDK for running embedded code. The SoC consists of the Processing System (PS) and the PL part. The PS with the ARM cores, caches, ports and controllers is connected to the PL with the IPs provided by Xilinx as illustrated in Figure 6. The IPs required for SoC design are Xilinx proprietary AXI protocol interconnects for AXI buses, AXI timer, the AXI Direct Memory Access (DMA) and a PS reset module. The AXI stream interface allows the hardware accelerator to connect to the PS through the AXI DMA IP. The DDR memory has higher throughput than the L2 cache and L2 cache provides lower latency. Due to low latency requirements associated with a real-time classification task, our SoC design connects the DMA to the L2 cache through the Accelerator Coherency Port (ACP). The ACP connects to the L2 cache via the Snoop Control Unit (SCU) which keeps all reads and writes coherent between the accelerator and the ARM cores. A number of synthesis optimizations were utilised for design optimisations.

The major parts of the design were implemented in the C/C++ high level programming language. The algorithm implementation in C/C++ is synthesized in Xilinx Vivado HLS design suite to verify the timing and design parameters, such as operating clock frequency and design latency. The high level code is then further optimised by manual code restructuring/rewriting and introduction of HLS optimization directives such as PIPELINE, LOOP UNROLL etc. The synthesis parameters are again verified for timing and design parameters, until satisfactory results are achieved.

### 7.1. Hardware Acceleration

**Figure 7:**
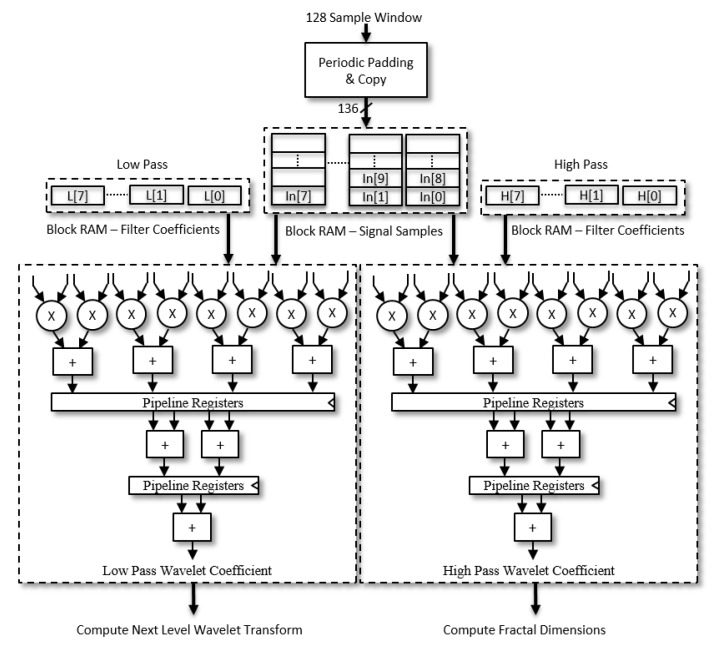
Hardware arithmetic pipeline of wavelet transform.

*Multi-Level Wavelet Transform Hardware:* The low pass filter and high pass filter wavelet coefficients are calculated from the original signal with periodic padding and the process is repeated for 4-level wavelet transform. Next level wavelet transform is computed from the previous low pass wavelet coefficients. The final level-4 low pass wavelet coefficients are used as features in the classification, while the detail coefficients from each stage are used to determine the fractal dimensions. The hardware DWT implementation is based on signal convolution with filter coefficients and subsequent down sampling of signal values. Our implementation stores the filter coefficients in the reverse order in block RAM and does not require flipping the filter coefficients for convolution. It allows us to access 8 simultaneous signal samples as well as filter coefficients for Daubechies 4 wavelet from the block RAM partitioned into 8 blocks with two reading ports each. A single loop computes both set of wavelet coefficients low pass approximations and high pass details. The computation for every alternate coefficient is skipped instead of down sampling later to save computation time and power. The unoptimised and optimised DWT algorithms are illustrated in Algorithms 1 and 2, respectively. The computation of a single coefficient is done with an eight sum of products pipelined operation for low latency processing. The arithmetic tree for computation of wavelet transform is illustrated in Figure 7.

**Figure 8:**
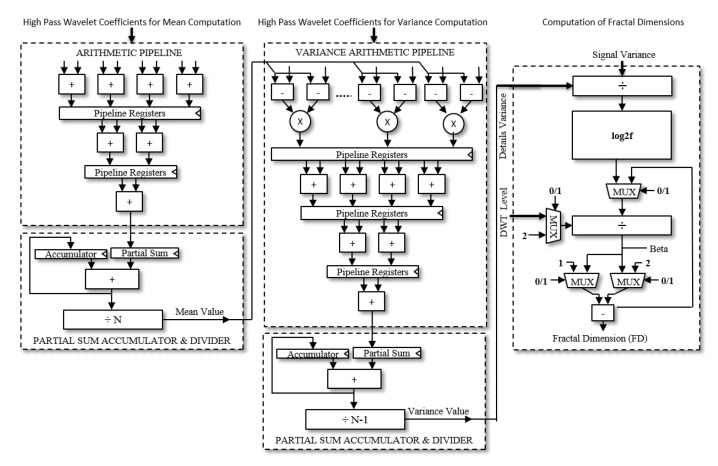
Logic circuit for mean, variance and fractal dimension computations.

*Fractal Feature Hardware:* The fractal dynamics is calculated for each level of wavelet detail coefficients. The fractal dimension is based on an an efficient log to the base 2 operation of the ratio of variance of wavelet detail coefficients to the original zero mean signal. The computation of mean and variance is performed through arithmetic trees and the fractal dimension is calculated with the synthesized circuit operations given in Figure 8. The algorithm is illustrated in Algorithm 3.

*LDA Classifier:* The coefficients obtained from LDA training are used in the classifier to evaluate a discriminant function between falls and no falls. The classifier is implemented in software on the processing system ARM core. The set of feature values are received from the hardware accelerator through the ACP port connected to the AXI bus. The features are read by the ARM core and the discriminant function is computed and the output is fed to a simple decision tree for a fall or a no fall decision, which determines if the value is ≥0 for a fall decision or not. The hardware accelerator is based on five design iterations. The final design is proposed as an efficient feature extraction accelerator for classification. The latency of the proposed arithmetic circuits varied from 8.6 to 9.2 nsec during the synthesis phase, hence a clock period of 10nsec, corresponding to the frequency of 100 MHz was utilised for the entire design. The designs with their optimizations and code restructuring are explained below:

- **Design I**: The design I consists of embedding the wavelet filter coefficients in local memory, to allow hardware to perform fast operations with low latency access to filter coefficients and reduced main memory access operations. The intermediate result arrays used in the algorithm are also embedded in local memory for fast read and write access. Furthermore, the functions are inlined to take advantage of synthesis optimizations with their surrounding code.
- **Design II**: The design II is further optimised by adding pipelines to the padding implementation, wavelet transform and variance calculation for computation of fractal dimensions.
- **Design III**: The design III consists of unrolling the loops over and above design II. The loops representing convolution operations in wavelet transform and variance computations are unrolled by a factor of 8.
- **Design IV**: The design IV is implemented with arithmetic trees. It only assumes elements of design I for its implementation. The computations of wavelet transform and variance for fractal dimensions are resolved into tree structures. It requires code restructuring and rewriting. The loops are manual unrolled to accommodate computational arithmetic tree structures.
- **Final Design**: The final design is based on pipelining the arithmetic trees implemented in design IV and manual unroll. Along with the optimizations of design I and IV, here we propose the technique of skipping the alternative computations of the wavelet filter convolutions instead of downsampling after the convolution operation is performed. The downsampling is embedded in the convolution computation rather than implemented separately. The integration reduces latency and number of stages within the algorithm, resulting in savings to execution time, logic and memory resources.

**Algorithm 1:** Discrete Wavelet Transform Algorithm.

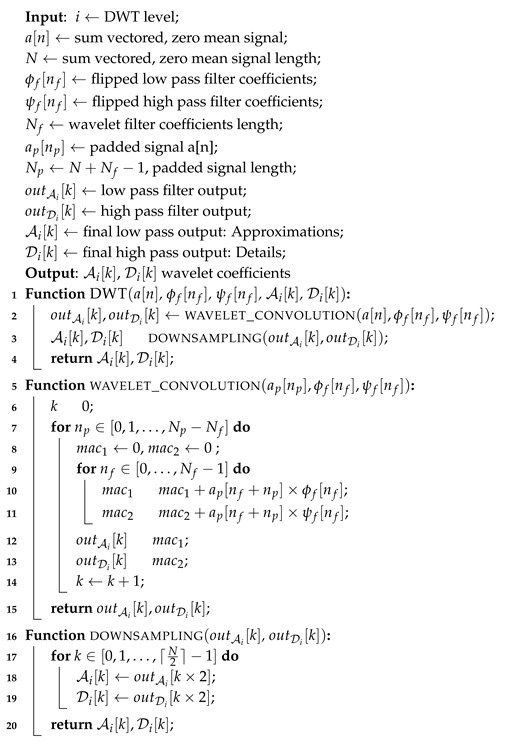



**Algorithm 2:** Proposed Optimised Discrete Wavelet Transform.

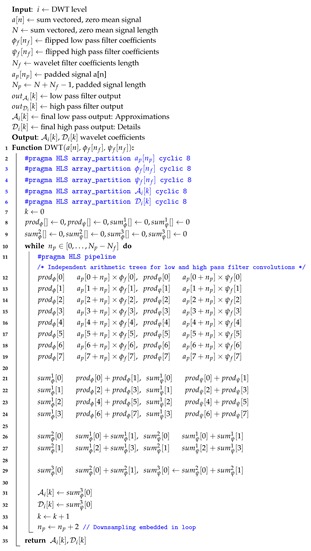



**Algorithm 3:** Proposed Fractal_Dim.

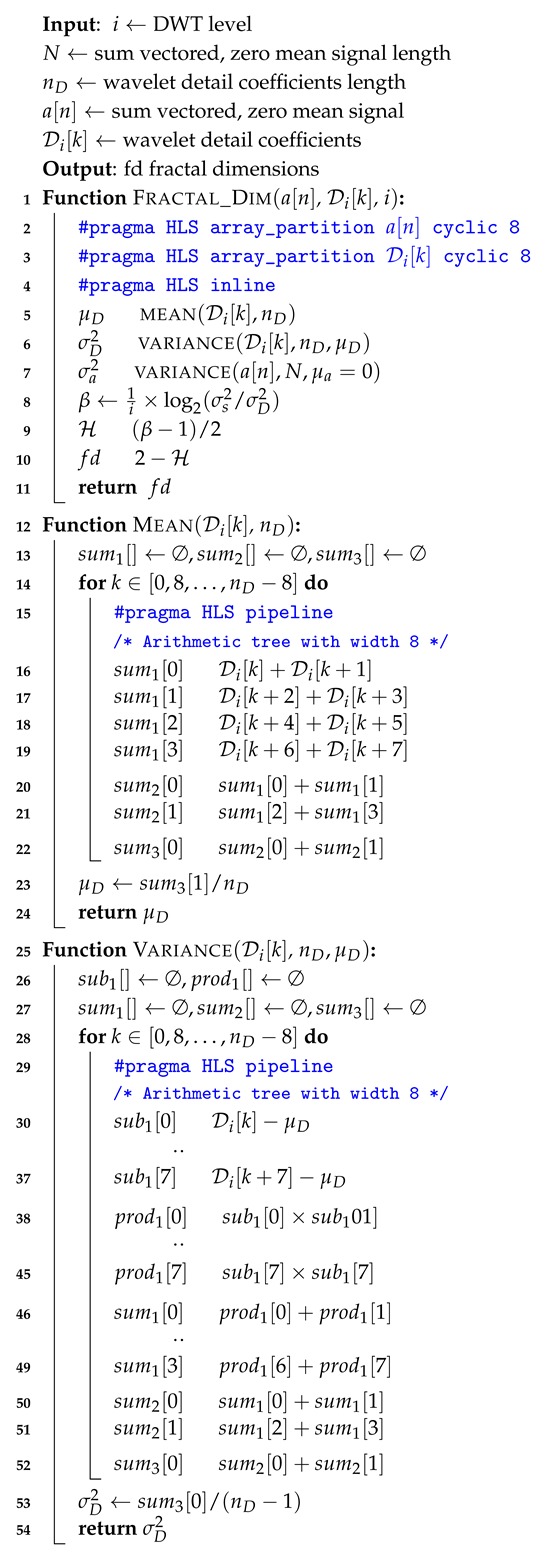



*Synthesis Results:* The resources required for the proposed hardware accelerators are given in Table 5. The final design uses around 3× more resources than the design I. However, the overall resource utilization remains relatively low at 28.67%, which is a significantly low value keeping in view a decrease of 10× in latency compared to design I and keeps logic area low for power efficient design.

## 8. Hardware System Results

**Figure 9:**
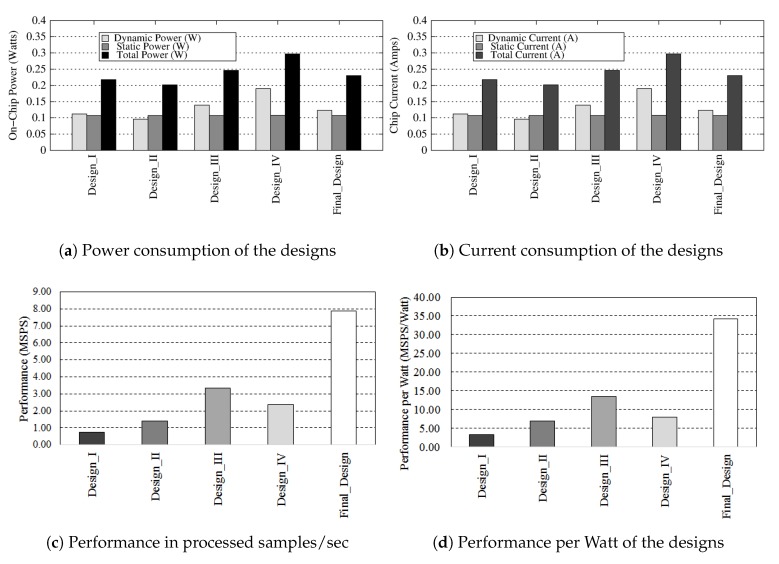
Power and performance results of hardware designs.

*Power Consumption*: The power consumption of the design iterations and the final design is given in Table 6. The final optimised design consumes 0.23 W power, an increase of 4.8% over design I with 10× improvement in latency. Furthermore, it shows an improvement of 6.52× over the power consumption of the ARM core at 666 MHz. The dynamic, static and total power of all designs is illustrated in Figure 9a, while the current intake of the hardware designs is shown in Figure 9b. The dynamic power consumption of the final design remains almost the same, however there is a small increase in static power consumption. The current consumption verifies this since there is an increase in static current.

**Figure 10:**
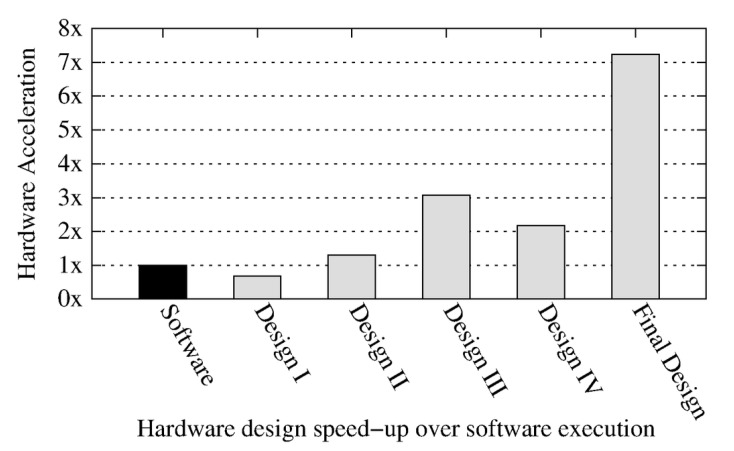
Hardware speed-up relative to software execution.

*Speed-Up*: The final design shows a speed-up of 7.3× over software execution as illustrated in Figure 10. The design III with pipelining and unrolling of loops in wavelet transform and variance computations gives around 3× improvement over software execution, however further improvements to design does not result in any considerable advantage but incurs high area overhead costs. The arithmetic tree design decision with clever optimizations to code structure and flow were deemed necessary for good results.

*Performance & Performance per Watt*: The performance of the design in Million Samples Processed per Second (MSPS) is shown in Figure 9c and performance per Watt in MSPS per Watt is illustrated in Figure 9d. The final design has the highest performance at 7.86 MSPS, an improvement of 10.68× over design I and a performance per Watt of 34.16 MSPS per Watt, an improvement of 10.11× over design I. The improvements in performance per Watt over software execution are 47.6×.

The proposed FDS is compared with current and comparable work in Table 7. The proposed FDS demonstrated the highest accuracy, sensitivity and precision with 99.38%, 99.10% and 99.96%, respectively. The proposed system has the lowest latency of 0.1 μs/sample compared to all the given systems. Our system achieves the lowest latency of 0.1 μs/sample by utilising pipelined arithmetic tree structures, which can process an entire window segment of 128 samples in 1629 cycles at 100 MHz operating frequency. The processing of entire window segment with arithmetic trees results in a higher throughput and an effective latency of 0.1 μs/sample with 128 sample bursts of data streaming into the proposed hardware design. The latest works in [66,67] have a higher latency of 1 s, while [47,50,51] have latencies in microseconds, however they suffer form lower classification performance. Furthermore, the proposed system also consumes relatively low to moderate resources over all hardware implementations from design I to final design, which allows flexibility in performance and area trade-off requirements for processing. The final design consumes lower LUT resources than [67,68], and final optimised design in [47] with 17.63%, 18.43% and 26.29% of the LUT resource consumption of the designs, respectively. While our final design consumes higher LUT resources at 167% and 292% of [50,51] respectively, but has 10% higher sensitivity and 2.9% higher specificity than both the designs. The first design iteration design I of our proposed system can be utilised for low area/resource consumption and consumes the second lowest LUT resources of all the designs in Table 7 with only [51] consuming 2% less resources. Design II also consumes low resources in Table 7 with [51] consuming 10.9% less resources. Design III and IV are placed in the middle of Table 7, in terms of LUT resource consumption, lower than [67,68], but higher than [50,51].

Similarly, the proposed design consumes lower number of signal processing blocks DSP48E at 10, while the other designs in [51,68] and the final optimised design in [47] consume 12, 28 and 70 blocks, respectively. The unoptimised initial design in [47], however consumes half the number of signal processing blocks but suffers from lower fall detection performance. The proposed design consumes higher RAM blocks in order to simultaneous provide data samples and filter coefficients to pipelined arithmetic trees, which results in the lowest latency of all designs.

All of the current and comparable works in Table 7 except the proposed system suffer from lack of reproducible classification results. The classification performance and accuracies of the proposed system in Table 7 are reproducible due to validation through a public dataset, while all the remaining works lack reproducibility of results due to the use of self-simulated experiments for which data is not available publicly. While the system in [67] also utilises the Mobifall dataset [26], however the dataset is combined with self-simulated experiments and provides combined results which are not reproducible.

## 9. Conclusions

This work presents the hardware/software co-design of a novel fall detection system based on fractal features for learning and classification. Fractal dimensions capture irregularity characteristics of the signal and provide good discriminant capability for falls against ADLs with a high classification accuracy of 99.38%. While, wavelet transforms can be used to compute fractal dimensions of non-stationary signals, the process is computationally intensive. An embedded wearable SoC for our proposed fall detection based on fractal features can provide higher accuracy at low performance per Watt through reconfigurable design innovations. The computationally intensive wavelet transform and fractal dimension computations are accelerated on reconfigurable hardware through clever design optimizations, while LDA based machine learning algorithm is implemented in parallel on the ARM Cortex A9 chip. The 100 MHz design frequency provided high throughput and low latency with a good trade-off between performance and power consumption, which was sufficient for our case. The final hardware design gives 7.3× speed-up and 6.53× improvements in power consumption, compared to the software only execution. The final design also provides the lowest latency of 0.1 μs/sample processed as compared to all the current FPGA based implementations of FDS. Overall the performance per Watt yields advantage of 47.6×. The proposed FDS provides high throughput with a high classification accuracy, sensitivity and precision of 99.38%, 99.10% and 99.96% respectively. Additionally, the classification performance of the proposed system is reproducible due to validation through public dataset, unlike current embedded FPGA implementations of FDS which utilise self-simulated experiments. Moreover, the proposed FDS consumes relatively low programmable logic resources of the Zynq device at 28.67%. Furthermore, the achieved performance provides the sustainability and scalability for multiple multichannel sensors for future work.

## Figures and Tables

**Table 1:** ARFIMA model for falls, walking and picking up objects.

Activity	ARFIMA	AR(1)	MA(1)/MA(4)	Likelihood	AIC	*d*	$\tilde{d}$	*H*
Falling	(1,d,1)	0.64	0.46	1144.5	−3.94	0.49	1.49	0.99
Walking	(1,d,4)	0.86	2.1, 2.3, 1.9, 0.8	2785	−9.27	−0.48	0.52	0.02
Picking up	(1,d,4)	0.9	1.5, 1.6, 1.2, 0.4	2353	−10.13	−0.32	0.68	0.18

**Table 2:** Hurst exponent from ARFIMA analysis.

Activity	*d*		$\tilde{d}=d+1$	H=d+0.5	fd=2−H
	Mean	SD	Mean	Mean	Mean
Falls	1.490	0.003	1.49	0.99	1.01
Walking	0.518	0.002	0.518	0.018	1.982
Kneeling down	0.554	0.258	0.554	0.054	1.946
Sitting down	0.999	0.000	0.999	0.499	1.501
Standing up	0.99	0.007	0.990	0.490	1.51
Picking up objects	0.699	0.091	0.699	0.199	1.801
Lying	0.225	0.347	-	-	-

**Table 3:** Classification accuracy for features.

Features	LDA Classifier
	Accuracy (%)
Wavelet Transform Lvl 2	79.77 (±0.08)
Wavelet Transform Lvl 3	83.09 (±0.08)
Wavelet Transform Lvl 4	85.76 (±0.09)
Fractal Features	91.84 (±0.21)
Fractal, Wavelet Lvl 4	95.61 (±0.09)
Fractal, Wavelet Lvl 4, Mean, SD	99.38 (±0.19)

**Table 4:** Classification Performance Comparison.

Authors	KSE’18 [60]	IEEE Sensors’18 [61]	PEIS’19 [64]	IEEE Access’19 [62]	IEEE Sensors’19 [63]	Proposed FDS
Dataset	Self-simulated	SisFall Data [65]	Fall Data [8]	Public Datasets	SisFall Data [65]	Fall Data [8]
Sensor	Tri-axes Acc.,	Tri-axes Acc.	Tri-axes Acc.	Tri-axes Acc.,	Tri-axes Acc.,	Tri-axes Acc.
Sensor Location	Gyro.			Gyro.	Gyro.	Pelvis
	Hip	Waist, Chest,	Waist	Thigh, Chest	Waist	
Features	Mean, SD,	Mean, SD,		Mean, Maxima,	Mean, Maxima,	
	Energy, Entropy,	Maxima, Minima,	x, y, z Axes	Minima, Auto	Minima, SD,	Fractal and
	Hjorth Mobility	Kurtosis,	Acceleration	Cross Correlation	Sum Vector,	Wavelet Lvl
	and Complexity,	Skewness, Corr.		Peak PSD etc.	Kurtosis,	4 Features,
	Sum Vector etc.	Coefficients etc.			Skewness etc.	Mean, SD
Classifier	SVM, RF	SVM, KNN, DT,	Deep Hybrid	ANN, KNN,	KNN, SVM,	LDA
		Naive Bayes	RNN	EBT, QSVM	RF	
Sensitivity	94.37%	98.30%	-	-	80.07%	99.10%
Specificity	-	-	-	-	98.27%	99.90%
Accuracy	-	97.60%	92.23%	97.70%	96.82%	99.38%

**Table 5:** Hardware designs resource utilization and latency.

Hardware Resources	Design I		Design II		Design III		Design IV		Final Design	
	Used	%	Used	%	Used	%	Used	%	Used	%
LUT Logic	2516	4.73	2775	5.22	4394	8.26	5403	10.16	7222	13.58
CARRY4	141	1.06	141	1.06	139	1.05	139	1.05	139	1.05
Register	3328	3.13	3637	3.42	7370	6.93	10,161	9.55	12,853	12.08
LUT Shift Reg.	112	0.64	121	0.7	1419	8.16	132	0.76	147	0.84
LUT Dist. RAM	160	0.92	160	0.92	224	1.29	288	1.66	192	1.1
Muxes	10	0.02	10	0.02	15	0.03	10	0.02	10	0.02
Total	6902	10.5	7459	11.34	14,265	25.72	17,353	23.2	21,649	28.67
Block RAM	7	5	7	5	8.5	6.07	10	7.14	12	7.14
DSP48E	10	4.55	10	4.55	10	4.55	10	4.55	10	4.55
I/O	119	59.5	119	59.5	119	59.5	119	59.5	119	59.5
Latency cycles at 100 MHz	17381		9070		3841		5423		1629	

**Table 6:** Hardware designs power consumption.

Hardware Resources	Design I	Design II	Design III	Design IV	Final Design
	(Watts)	(Watts)	(Watts)	(Watts)	(Watts)
Clocks	0.029	0.029	0.044	0.056	0.047
LUT Logic	0.013	0.014	0.019	0.025	0.015
CARRY4	0.001	0.001	0.001	0.001	<0.001
Register	0.001	0.001	0.002	0.003	0.001
LUT Shift Reg.	<0.001	<0.001	<0.001	<0.001	<0.001
LUT Dist. RAM	<0.001	<0.001	<0.001	<0.001	<0.001
Muxes	<0.001	<0.001	<0.001	<0.001	<0.001
Total	0.016	0.016	0.022	0.03	0.017
Signals	0.022	0.022	0.037	0.052	0.029
Block RAM	0.02	0.013	0.018	0.026	0.016
DSP48E	0.006	0.006	0.006	0.005	0.002
I/O	0.019	0.011	0.012	0.02	0.012
Static Power	0.107	0.107	0.107	0.108	0.107
Total Power	0.218	0.202	0.246	0.297	0.23

**Table 7:** Proposed algorithm system comparison with current comparable work.

	AICCSA’14 [47]	SAI’16 [68]	AICCSA’16 [50]	IDT’16 [51]	BHI’17 [66]	TBioCAS’19 [67]	Proposed System
Sensor	Tri-axes Acc.	ECG + Acc.	Tri-axes Acc.	Tri-axes Acc.	Tri-axes Acc.	Tri-axes Acc.	Tri-axes Acc.
Method	PCA + DT	K-NN	Thresholding and SVM	Thresholding and SVM	Thresholding	Thresholding	Fractal Features
Processor	Zynq	Zynq Z-7000	Zynq Z-7010	Zynq Z-7010	FPGA	Virtex5	Zynq Z-7020
Frequency	121–298 MHz	100 MHz	-	-	-	1 KHz	100 MHz
Latency	97.86–2 μs	-	0.86 μs	6.34 μs	1 s	1 s	0.1 μs/sample
LUT	3381–27465	40955	4314	2470	-	39190	2516–7222
Flip Flops	1352–10129	24015	3815	2539	-	21750	3328–12853
BRAM	4–0	2	1	2	-	-	12
DSP48E	5–70	28	-	12	-	-	10
Validation	Self-simulated	Self-simulated	Self-simulated	Self-simulated	Self-simulated	Self-simulated	Fall Dataset [8]
Reproducibility	x	x	x	x	x	x	
Sensitivity	-	-	89.50%	89.50%	98.10%	98.60%	99.10%
Specificity	-	-	97.00%	97.00%	99.20%	99.10%	99.90%
Accuracy	88.40%	82.14%	-	-	-	-	99.38%

## Mathematical Notation

a	Sum vectored, zero mean acceleration signal vector
$a^{\prime }$	Sum vectored, acceleration signal vector
$a_{x}$	Acceleration signal vector along x-axis
$a_{y}$	Acceleration signal vector along y-axis
$a_{z}$	Acceleration signal vector along z-axis
a(n)	Acceleration signal at sample *n*
$A_{i}$	Wavelet approximation coefficients at level *i*
β	Signal spectral exponent
*d*	Fractional difference parameter
$\tilde{d}$	Fractional integration coefficient
$d_{c}$	Drift constant in stationary test
$D_{i}$	Wavelet detail coefficients at level *i*
e(n)	Innovation process in stationary test
ϵ(n)	Random variable
$f_{s}$	Sampling frequency
fd	Fractal dimension
γ	Autoregressive coefficient with value < 1
Γ	Gamma Function
*H*	Hurst exponent
$H_{0}$	Null hypothesis in stationary test
$H_{1}$	Alternative hypothesis in stationary test
*i*	Wavelet transform level
I(d)	Integrated part of ARIMA/ARFIMA model
*k*	Shift/translation index
L	Lag operator
λ	Deterministic trend with coefficient
$\mu_{a}$	Mean of sum vectored, zero mean acceleration signal vector
$\mu_{a^{\prime }}$	Mean of sum vectored acceleration signal vector
$\mu_{D_{i}}$	Mean of wavelet detail coefficients at level *i*
$\mu_{s}$	Mean of accelerometer first order difference signal
*n*	Signal sample number
*N*	Total number of samples
ω	Angular frequency
p-value	Confidence value for unit root tests
$p_{max,ADF}$	Maximum delay value for ADF test
$p_{max,KPSS}$	Maximum delay value for KPSS test
$\phi_{i,k}\left(n\right)$	Scaling wavelet function with shift index *k*, at level *i*
$\psi_{i,k}\left(n\right)$	Mother wavelet function with shift index *k*, at level *i*
*q*	Number of random perturbations
*r*	Number of autoregressive terms
s(n)	First order difference, accelerometer signal
S(ω)	Power spectrum density
$\sigma_{a}^{2}$	Variance of sum vectored, zero mean acceleration signal vector
$\sigma_{a^{\prime }}^{2}$	Variance of sum vectored acceleration signal vector
$\sigma_{D_{i}}^{2}$	Variance of wavelet detail coefficients at level *i*
$\theta_{p}$	General moving average coefficients
$v_{1}\left(n\right)$	Stationary process
$v_{2}\left(n\right)$	Independent and identically distributed process with 0 mean
$\zeta_{r}$	General autoregressive coefficients
